# Gut Microbiota-Derived Trimethylamine Promotes Inflammation with a Potential Impact on Epigenetic and Mitochondrial Homeostasis in Caco-2 Cells

**DOI:** 10.3390/antiox13091061

**Published:** 2024-08-30

**Authors:** Laura Bordoni, Irene Petracci, Giulia Feliziani, Gaia de Simone, Chiara Rucci, Rosita Gabbianelli

**Affiliations:** 1 Unit of Molecular Biology and Nutrigenomics, School of Pharmacy and Health Products, University of Camerino, 62032 Camerino, Italy; irene.petracci@unicam.it; 2 School of Advanced Studies, University of Camerino, 62032 Camerino, Italy; giulia.feliziani@unicam.it (G.F.); gaia.desimone@unicam.it (G.d.S.); chiara.rucci@unicam.it (C.R.)

**Keywords:** nutrigenomics, inflammation, trimethylamine, mitochondria, epigenetics

## Abstract

Trimethylamine (TMA), a byproduct of gut microbiota metabolism from dietary precursors, is not only the precursor of trimethylamine-N-oxide (TMAO) but may also affect gut health. An in vitro model of intestinal epithelium of Caco-2 cells was used to evaluate the impact of TMA on inflammation, paracellular permeability, epigenetics and mitochondrial functions. The expression levels of pro-inflammatory cytokines (IL-6, IL-1β) increased significantly after 24 h exposure to TMA 1 mM. TMA exposure was associated with an upregulation of SIRT1 (TMA 1 mM, 400 μM, 10 μM) and DNMT1 (TMA 1 mM, 400 µM) genes, while DNMT3A expression decreased (TMA 1 mM). In a cell-free model, TMA (from 0.1 µM to 1 mM) induced a dose-dependent reduction in Sirtuin enzyme activity. In Caco-2 cells, TMA reduced total ATP levels and significantly downregulated ND6 expression (TMA 1 mM). TMA excess (1 mM) reduced intracellular mitochondrial DNA copy numbers and increased the methylation of the light-strand promoter in the D-loop area of mtDNA. Also, TMA (1 mM, 400 µM, 10 µM) increased the permeability of Caco-2 epithelium, as evidenced by the reduced transepithelial electrical resistance values. Based on our preliminary results, TMA excess might promote inflammation in intestinal cells and disturb epigenetic and mitochondrial homeostasis.

## 1. Introduction

Trimethylamine (TMA) is an amine generated in the colon by the gut microbiota through the processing of nutritional substrates like choline, betaine, L-carnitine, and dimethylglycine and their precursors (phosphatidylcholine, crono-betaine, and g-butyrobeaine) [1,2]. These substrates are commonly found in red meat, fish, eggs, and related sources. TMA generated in the gut is absorbed through passive diffusion and then transported via the portal circulation to the liver, where it is oxidized into trimethylamine-N-Oxide (TMAO) by hepatic flavin monooxygenases (FMOs) [3]. TMAO is a low-molecular-weight compound which has been associated with the development of several chronic non-communicable diseases and proposed as a possible biomarker of cardiovascular diseases (CVDs) [4]. Despite a body of literature about the potential harmful effects of TMA and TMAO, evidence on this topic is still fragmentary. Although being a well-known uremic toxin, TMA has been understudied concerning its effects on human health, largely due to the predominant focus on its derivative, TMAO. While it has been suggested that TMA may pose significant health risks [5], only a limited number of studies have delved into the mechanistic effects of TMA on health. Jalandra and collaborators showed that TMA has toxic effects in vitro, with acute exposure leading to decreased cell viability and ATP production in HCT116 and HT29 cells [5]. In this model, TMA induces oxidative stress by increasing cellular superoxide production [5], potentially impacting mitochondrial dynamics, although the precise mechanism remains unknown. Additionally, intrarectal and intraperitoneal injections of TMA in FVB/J mice led to a significant increase in inflammatory cell infiltration in the colon and rectum, also supporting the idea of the local pro-inflammatory action of TMA in vivo [5].

However, due to the limited literature on this topic, the exact mechanism by which TMA promotes inflammation is still unclear. Nonetheless, recent evidence suggests that TMA-induced inflammation may result from the perturbation of mitochondrial homeostasis [5].

Indeed, the connection between inflammation and mitochondrial functions is well documented [6]: by releasing reactive oxygen species during aerobic respiration, mitochondria can contribute to oxidative stress, thereby sustaining inflammation and causing cellular damage. Simultaneously, inflammation disrupts mitochondrial dynamics and function, promoting the production of reactive oxygen species [6].

Moreover, the mitochondrial damage induced by inflammation may also manifest with an aberrant mitochondrial DNA copy number (mtDNAcn), which is defined as the number of copies of mitochondrial DNA present in a cell. The maintenance of an appropriate mtDNAcn in the cell is essential for the regular expression of mitochondrial proteins, which overall control and determine the mitochondrial function [7]. For this reason, mtDNAcn has been considered a proxy indicator for mitochondrial activity and a biomarker of mitochondrial health [7,8]. New conjectures propose the potential influence of mtDNA methylation on mitochondrial functions, especially in the context of cardiovascular and metabolic diseases [9,10]. Notably, studies have measured DNA methylation in the displacement loop (D-loop) of mtDNA, a region that being crucial for mtDNA replication and transcription, might also affect mtDNAcn [11]. Furthermore, mtDNA methylation has been hypothesized to be linked to multifactorial diseases (such as obesity and metabolic diseases) and to environmental exposures such as diet [10,12,13,14]. However, this remains a contentious topic, as certain evidence contradicts the presence of the methylation on mtDNA, despite the identification of enzymes accountable for DNA methylation within mitochondria [15].

Epigenetic regulations can also affect inflammation: epigenetic mechanisms are responsible for the regulation of inflammatory genes’ expression, but in turn, inflammation can affect chromatin remodeling and epigenetic enzymes activity [16,17]. Among the numerous enzymes involved in these complex regulations, DNA methyltransferases (DNMTs) (major determinants of DNA methylation) and Sirtuins (SIRTs) (histone deacetylases connected with both nuclear and mitochondrial functions) are central mediators linking epigenetic regulations to inflammation, mitochondrial dysfunctions and oxidative stress [18,19,20,21,22,23,24,25].

Lastly, mitochondrial dysfunction may compromise the ATP production system and alter the mitochondrial membrane potential (Δψm) of the cell [26,27,28]. Changes in the mitochondrial membrane potential trigger apoptotic signaling pathways that culminate in the release of mitochondrial components, including mtDNA, into the extracellular environment, potentially fostering inflammation [8,29,30,31].

This study aims at testing the hypothesis that disturbances in ATP production and mitochondrial membrane potential, as well as alterations of mtDNAcn and cell-free mitochondrial DNA (cf-mtDNA) released can be induced by TMA exposure in an intestinal model of Caco-2 cells. Additionally, given the well-established association between inflammation and epigenetic homeostasis, we aimed at testing the capacity of TMA to affect the expression levels and activity of DNMTs and SIRTs—pivotal enzymes in the epigenetic landscape.

## 2. Materials and Methods

### 2.1. Cell Culture

To investigate the impact of TMA on intestinal cells, Caco-2 cells, which are a human colonic epithelial cell line (ATCC, Rockville, MD, USA), were selected. Cells were provided by Professor Ivan Nabissi, University of Camerino. Caco-2 cells were cultured in Dulbecco’s modified Eagle’s medium (DMEM) supplemented with 10% heat-inactivated fetal bovine serum (FBS), 1% L-glutamine, 1% non-essential amino acids (NEAAs), and 1% penicillin/streptomycin. The cells were maintained at 37 °C in a humidified atmosphere with 5% CO_2_. The medium was refreshed every 2 days, and cells were passaged when reaching 80% confluence.

In addition, to test the effect of TMA on intestinal permeability, these cells were cultured in a transwell-based system, as described by Kyeong Jin Kim et al. [32]. Briefly, Caco-2 cells were seeded on non-coated transwell inserts (0.4 µm pore size, ThinCert^®^, Greiner Bio-one, Frickenhausen, Germany) at the density of 1 × 10^5^ cells/insert in a 6-well plate and maintained in complete medium. The culture medium was added in both apical (AP) and basal (BL) compartments and replaced every 2 days. The 6-well plates were incubated in atmosphere of 5% CO_2_ at 37 °C. Cells were cultured in transwells until differentiation and complete epithelium formation. In order to assess the evolution of the intestinal epithelium formation, the transepithelial electrical resistance (TEER), which indicates the integrity of Caco-2 epithelium, was measured using Millicell^®^ ERS (Millipore, Merk, Darmstadt, Germany) after 4, 7, 10, 14, and 17 days post seeding.

### 2.2. Cell Viability Assay

The cytotoxic impact of TMA was assessed through the 3-(4,5-Di-2-yl)-2,5-ditetrazolium bromide (MTT) assay (Thiazolyl blue tetrazolium bromide 98%, code 158990050, Acros Organic, Fair Lawn, NJ, USA). In brief, Caco-2 cells were plated in 96-well plates at a density of 1 × 10^4^ cells/well in complete medium and exposed to various concentrations of TMA (1 nM, 10 nM, 0.1 μM, 1 μM, 10 μM, 100 μM, 1 mM, 10 mM, 100 mM) for 24 h. Following the incubation period, the cells were treated with a 5 mg/mL MTT solution. After four hours, the absorbance was measured at 540 nm using a spectrometer reader (FLUOstar Omega, BMG LABTECH’s, Ortenberg, Germany). The experiment was performed in biological quadruplicates.

### 2.3. Treatments

Caco-2 cells were exposed to TMA at concentrations of 10 µM, 400 µM, and 1 mM (directly solved in complete medium as a vehicle) for a duration of 24 h. The selection of the lowest TMA concentration was based on average physiological fecal TMA concentrations [33]. The other two concentrations tested (400 µM and 1 mM) were determined by the MTT assay results: TMA 1 mM represented the highest non-cytotoxic concentration, while TMA 400 μM was chosen as an intermediate value between 10 μM and 1 mM. A negative control (vehicle only) and two positive controls, lipopolysaccharide (LPS), at 100 ng/mL and 10 µg/mL, were included in the experimental settings. Experiments were conducted in triplicate. After 24-h incubation, both cells and medium were collected. The cell pellets were promptly frozen in liquid nitrogen and stored at −80 °C for subsequent analysis. The cell medium was centrifuged at 13,248× *g* for 5 min, and the clear supernatant was aliquoted into new nuclease-free conical tubes, then stored at −80 °C for future use.

To evaluate the inflammatory activity of TMA on the intestinal epithelium model, cells previously differentiated and grown in transwells were treated as follows: TMA at different concentrations (1 mM, 400 µM, and 10 µM) was added to the AP compartment and cells were incubated for 24 h. Two positive controls for inflammatory conditions, LPS 100 ng/mL and 10 µg/mL, were also included. The treatments were performed in biological triplicates.

### 2.4. Assessment of DNMT and SIRT Activities after Exposure to TMA

The nuclear protein fraction was isolated from Caco-2 cells using the Nuclear Extraction kit (Abcam, Waltham, MA, USA) according to the manufacturer’s instructions. Briefly, untreated Caco-2 cells were first lysed and then the nuclear protein fraction was isolated from the cytoplasmic fraction. The protein concentration of the nuclear extract was quantified by Bradford Assay, using bovine serum albumin (BSA) as a calibrator. The nuclear extract was used to evaluate the activity of DNMTs and SIRTs. The effect of TMA on DNMTs activity was evaluated using the EpiQuik™ DNA Methyltransferase Activity/Inhibition Assay Kit (EpigenTek, Farmingdale, NY, USA) according to manufacturer’s instructions. Briefly, 5 μg of nuclear proteins were transferred to a 96-well plate and incubated with various concentrations of TMA (1 mM, 400 μM, 100 μM, 50 μM, 10 μM, 1 μM, 100 nM, 10 nM, 1 nM, 0.01 nM, 0.001 nM, 0.000001 nM) for 120 min at 37 °C. The absorbance was measured at 450 nm using a microplate reader (FLUOstar Omega, BMG LABTECH’s, Ortenberg, Germany), and DNMT activity (OD/h/mg) was calculated.

The effect of TMA on SIRT activity was evaluated using the Universal SIRT Activity Assay Kit (Abcam, Waltham, MA, USA) following the manufacturer’s guidelines. In brief, 4 μg of nuclear proteins were transferred to a 96-well plate and incubated with various concentrations of TMA (1 mM, 400 μM, 100 μM, 50 μM, 10 μM, 1 μM, 100 nM, 1 nM, 0.01 nM, 0.001 nM, 0.000001 nM) for 90 min. The absorbance was recorded at 450 nm and SIRT activity (OD/min/mg) was calculated. The experiment was performed in technical duplicates.

### 2.5. MtDNAcn Quantification

Genomic DNA was extracted from Caco-2 cell pellets using DNAzol Reagent (Invitrogen, Carlsbad, CA, USA). Briefly, the cell pellets were lysed with DNAzol, 100% ethanol was added to the lysate to precipitate genomic DNA and two washes in 75% ethanol were performed to remove any contaminants from the isolated DNA.

Cf-DNA was extracted from the culture medium of treated Caco-2 cells using the Plasma/Serum Cell-Free Circulating DNA Purification Kit (Norgen, Biotek, Thorold, ON, Canada) according to manufacturer’s instructions. The concentration and purity of genomic DNA was measured using NanoDrop spectrophotometer (Thermo Fisher Scientific, Waltham, MA, USA) while the concentration of cf-DNA was assessed using Qubit Fluorometer (Thermo Fisher Scientific, Monza, Italy).

Genomic DNA was used to quantify relative mtDNAcn by quantitative Real Time Polymerase Chain Reaction (qPCR) (CFX connect, Biorad, Hercules, CA, USA) while cf-DNA was used to perform both relative and absolute quantification of cf-mtDNA and cell-free nuclear DNA (cf-nDNA) by digital PCR (QIAcuity, Qiagen, Venlo, The Netherlands).

For relative quantification of mtDNAcn, mtDNA-tRNALeu (RefSeq accession number NC_012920.1) (Fw: 5′-CACCCAAGAACAGGGTTTGT-3′; Rv: 5′-TGGCCATGGGTATGTTGTTA-3′) and Beta 2 microglobulin (B2M) (RefSeq accession number NC_000015.10) primers (Fw: 5′-TGCTGTCTCCATGTTTGATGTATCT-3′; Rv: 5′-TCTCTGCTCCCCACCTCTAAGT-3′) were chosen to amplify mtDNA and nuclear DNA (nDNA), respectively. Each analysis was run in technical duplicate. The mitochondrial primers were previously validated for their specificity (unique amplification of mtDNA) and the absence of coamplified nuclear insertions of mitochondrial origin (NUMTs) [34].

### 2.6. MtDNA Methylation

Bisulfite pyrosequencing was used to evaluate the methylation level of the mtDNA in two areas of the D-loop region [the promoter of the heavy strand (HSP) and the promoter of the light strand (LSP)] and in the ND6 gene. To avoid potential confounding factor due to NUMTs, mtDNA was isolated from nDNA by means of magnetic beads (Agencourt AMPure XP, Beckman Coulter, Brea, CA, USA) that selectively bind to mtDNA, and nDNA-specific enzymatic digestion (Plasmid-Safe™ ATP-Dependent DNase, Lucigen Biosearch Technologies, Middlesex, UK). To improve the efficiency of bisulfite conversion, the solution enriched with mtDNA was linearized using BamHI enzyme and then, converted with sodium bisulfite using the EZ-96 DNA Methylation-Gold kit (Zymo Research, Orange, CA, USA). PCR amplification was performed using the PyroMark PCR kit (Qiagen, Venlo, The Netherlands) in a thermal cycling device (2720 Thermal cycler, Applied Biosystem, Waltham, MA, USA). The selected areas of the mtDNA were investigated for methylation as previously described (primer sequences available in the original manuscript by Sun et al.) [35]. Gel electrophoresis was used to check the amplification accuracy. Amplicons were pyrosequenced using the PyroMark Q24 device (Qiagen, Venlo, The Netherlands) [35]. Biological triplicates for each treatment were analyzed.

### 2.7. Gene Expression Analysis

Total RNA was extracted from Caco-2 cells with the Total RNA Purification Plus Kit (Norgen Biotek, Thorold, ON, Canada), according to the manufacturer’s instructions and quantified (NanoDrop, Thermo Fisher Scientific, Italy). 1 µg of RNA was retrotranscribed to cDNA using the PrimeScript RT-PCR Kit (Takara Bio, Göteborg, Sweden) and quantitative real-time PCR (Biorad CFX96) was used to perform the gene expression analysis using TB Green^®^ Premix Ex Taq™ (Takara Bio, Göteborg, Sweden). The amplification conditions were: 30 s at 95 °C (denaturation), 5 s at 95 °C (annealing) and 30 s at 60 °C (extension) repeated for 40 cycles. The expression levels of the target genes were normalized relative to β-actin, using the 2^–∆∆Ct^ method. Each analysis was run in technical duplicate. An inter-run calibrator sample was applied to adjust the results obtained from different amplification plates. The target genes analyzed from Caco-2 cells were the epigenetic genes DNA methyltransferase 1 (DNMT1), DNA methyltransferase-3A (DNMT3A), DNA methyltransferase-3B (DNMT3B), Sirtuin-1 (SIRT1), Sirtuin-6 (SIRT6), and Sirtuin-7 (SIRT7), the pro-inflammatory genes interleukin-6 (IL-6) and interleukin 1β (IL-1β), the mitochondrial genes NADH dehydrogenase 6 (ND6), Cytochrome B (CYTB), Cytochrome c oxidase 1 (CO1), ATP synthase membrane 6 (ATP6) and the thigh junctions genes Zonulin 1 (ZO-1), Occludin (OCLN) and Claudin (CLDN1). The sequences of the primers used in the study are listed in Table S1 of Supplementary Materials. Biological triplicates for each treatment were analyzed.

### 2.8. ATP Quantification

The ATP content from treated Caco-2 cells was quantified using the ATP Colorimetric Assay Kit (Sigma-Aldrich, Darmstadt, Germany) according to the manufacturer’s instructions. Briefly, pellets from treated Caco-2 cells were lysed, and the ATP content was determined by phosphorylating glycerol. To correct for background in samples, especially background caused by glycerol phosphate, we included a Sample Blank by omitting the ATP Converter, as detailed in the manufacturer’s instructions. The Sample Blank readings were then be subtracted from the sample readings. The absorbance was read at 570 nm with a spectrometer (FLUOstar Omega, BMG LABTECH’s, Ortenberg, Germany). An ATP calibration curve using specific standards was generated and used for accurate ATP quantification. All analyses were run in triplicate. The effects of TMA on ATP levels were compared to the negative control (cells not exposed to TMA).

### 2.9. Mitochondrial Membrane Potential

The mitochondrial membrane potential (ΔΨm) was assessed by the Mitochondrial Membrane Potential Kit (Sigma-Aldrich, Germany). This assay utilizes the cationic, lipophilic dye JC-10, which can distinguish between living and apoptotic cells based on variations in their ΔΨm. In brief, 8 × 10^4^ cells were seeded into a 96-well plate, and various concentrations of TMA (0.01 µM, 0.1 µM, 1 µM, 10 µM, 100 µM, 200 µM, 400 µM, and 1 mM) were added to the cells. Additionally, two positive controls (LPS at 100 ng/mL and 10 µg/mL) and a negative control (vehicle only) were included. The treated cells were then incubated for 24 h at 37 °C. Subsequently, the cells were exposed to the JC-10 dye at 37 °C for 60 min, and the fluorescence was measured at two wavelengths (red at λex = 490/λem = 525 nm and at λex = 540/λem = 590 nm) using a fluorometer (FLUOstar Omega, BMG LABTECH’s, Ortenberg, Germany). Each experimental condition was set up in technical duplicate.

### 2.10. Permeability Assay

The TEER measurement was performed in cells cultured in transwells at 0, 6, and 24 h post treatment with TMA at different concentrations (10 µM, 400 µM, and 1 mM) or LPS (100 nM, 10 µM). Three values were measured for each well, which were then averaged and expressed as percentages. Also, the Lucifer Yellow (LY) (Sigma-Aldrich Life Science, Burlington, MA, USA) assay was used to measure the permeability of the Caco-2 multilayer. Briefly, the medium from both the AP and BL compartments was collected 24 h after treatments and stored at −80 °C for further analysis. Caco-2 cells on the AP side were gently washed twice with Phosphate-Buffered Saline (PBS) with Ca^2+^ and Mg^2+^ (Corning, Glendale, AZ, USA). LY at a concentration of 100 µM (in PBS with Ca^2+^ and Mg^2+^) was introduced into the AP compartment, while PBS (with Ca^2+^ and Mg^2+^) was added to the BL compartment. Subsequently, 150 µL of the solution from both compartments were promptly transferred to a 96-well plate, and fluorescence readings were taken using a fluorometer (FLUOstar Omega, BMG LABTECH’s, Ortenberg, Germany) at λEx/λEm = 485/520 nm. These measurements were repeated at 30, 60, and 120 min after the addition of the LY solution in the AP. The cells were maintained at 37 °C in a humidified atmosphere containing 5% CO_2_ between each reading. All analyses were conducted in biological duplicate.

### 2.11. Statistics Analyses

Statistical analyses were performed by using SPSS (IBM SPSS Statistics for Windows, Version 24.0, Armonk, NY, USA) and R version 3.5.3 (R Core Team, Vienna, Austria). An ANOVA test with Bonferroni’s correction for multiple testing was used to compare the difference between group means. A *p*-value < 0.05 was considered significant throughout the study. Results are shown as means ± SD.

## 3. Results

### 3.1. Cell Viability

**Figure 1:**
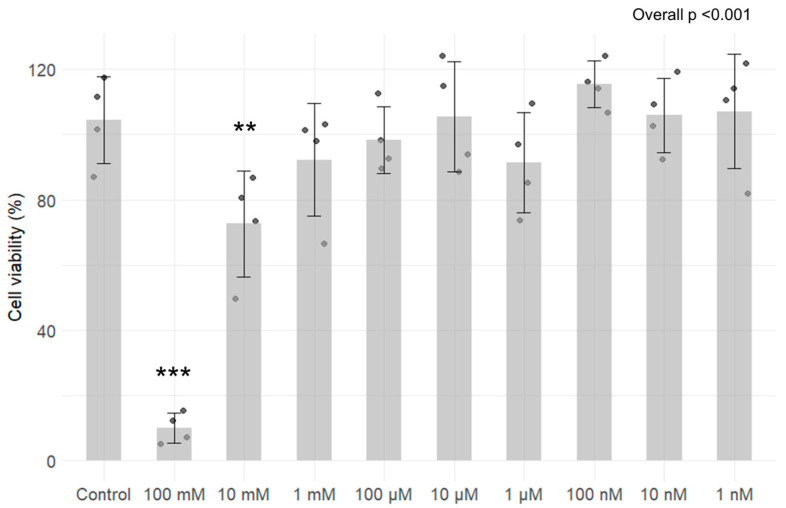
Assessment of cytotoxicity of TMA on Caco-2 cells after 24 h exposure to different concentrations (ranging from 100 mM to 1 nM). Cell viability is expressed in percentages with standard deviation for each condition. Results revealed that exposure for 24 h to TMA at 100 mM and 10 mM exhibit significant cytotoxicity. ** *p* < 0.01; *** *p* < 0.001 vs. Control.

The results from the MTT assay revealed that 24 h exposure to TMA at 100 mM (*p* < 0.05) and 10 mM (*p* < 0.05) concentrations exhibit significant cytotoxicity (Figure 1). Specifically, the residual viability of cells that were treated with 100 mM TMA is 10.01 ± 4.6%, while cells that were treated with 10 mM TMA show a viability of 72.66 ± 16.3%. No significant decrease in cell viability was measured for the other concentrations tested (Figure 1).

### 3.2. Expression Levels of Pro-Inflammatory Genes

**Figure 2:**
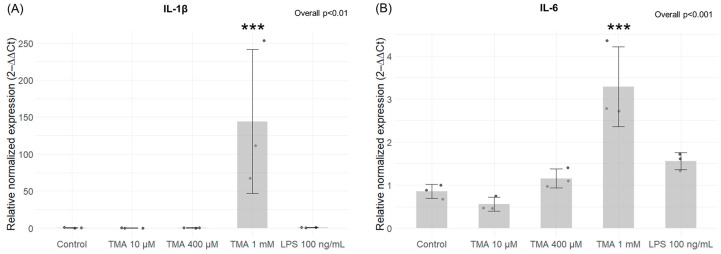
Expression levels of inflammatory genes. Expression levels of *IL-1β* (**A**) and *IL-6* (**B**) in Caco-2 cells measured by qPCR after TMA treatments at different concentrations (10 µM, 400 µM, and 1 mM) for 24 h. A statistically significant increase in the expression levels of both genes was measured in cells treated with TMA at 1 mM. *** *p* < 0.001 vs. Control.

Expression levels of the pro-inflammatory cytokines IL-1β and IL-6 were measured in Caco-2 cells exposed to various concentrations of TMA (10 µM, 400 µM, and 1 mM) and to LPS at 100 ng/mL. A statistically significant increase in the expression levels of both analyzed genes was measured in cells treated with TMA at 1 mM (IL-1β, *p* < 0.001; IL-6, *p* < 0.001), while no significant changes were observed at lower TMA concentrations (Figure 2A,B).

### 3.3. Expression Levels of DNMTs and SIRTs

**Figure 3:**
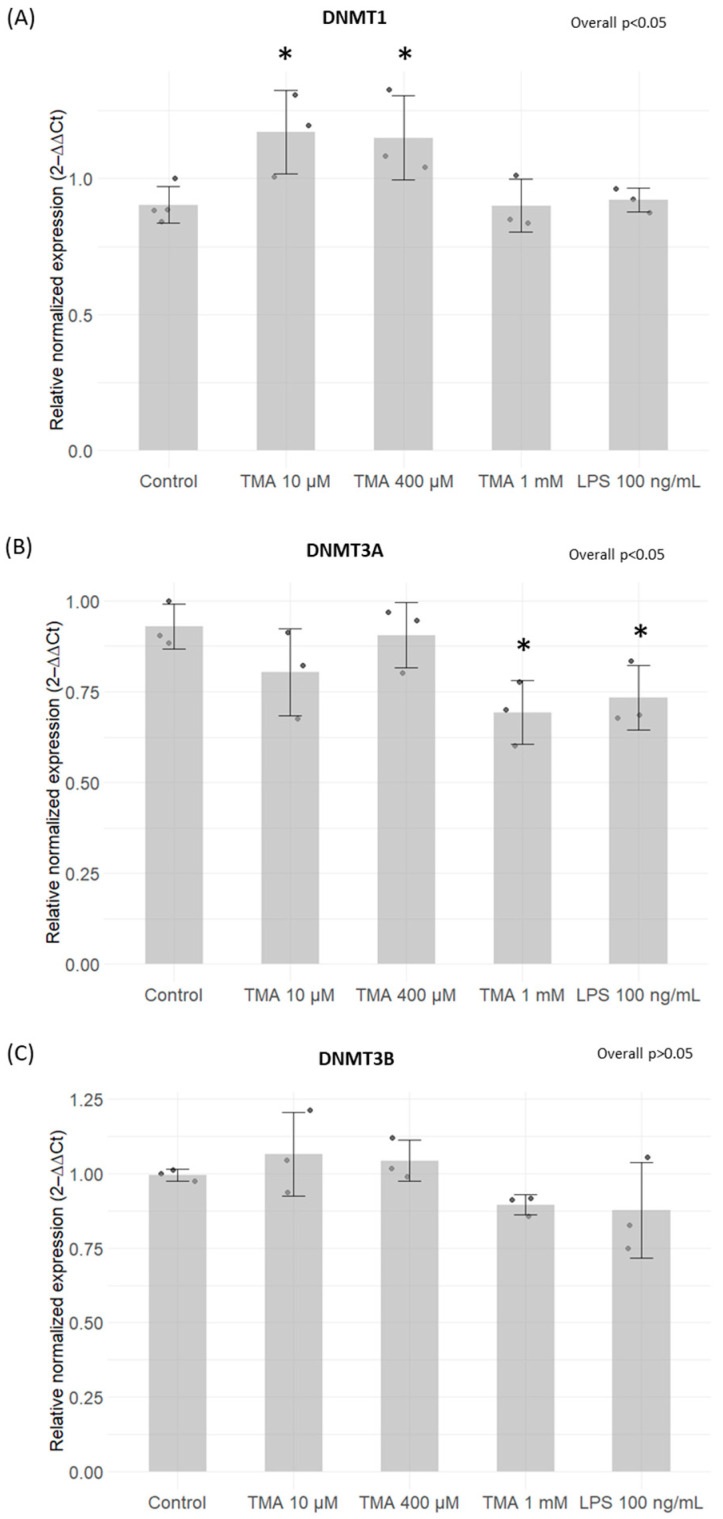
Expression levels of DNMTs measured by qPCR. *DNMT1* (**A**), *DNMT3A* (**B**) and *DNMT3B* (**C**) expression levels on Caco-2 cells after TMA treatments measured with the qPCR. A significant increase in DNMT1 expression was induced by TMA at 10 µM and 400 µM (**A**). A statistically significant decrease in DNMT3A expression was observed after exposure for 24 h to TMA at 1 mM and LPS at 100 ng/mL (**B**). DNMT3B expression remained unaffected by both TMA and LPS treatments (**C**). * *p* < 0.05 vs. Control.

**Figure 4:**
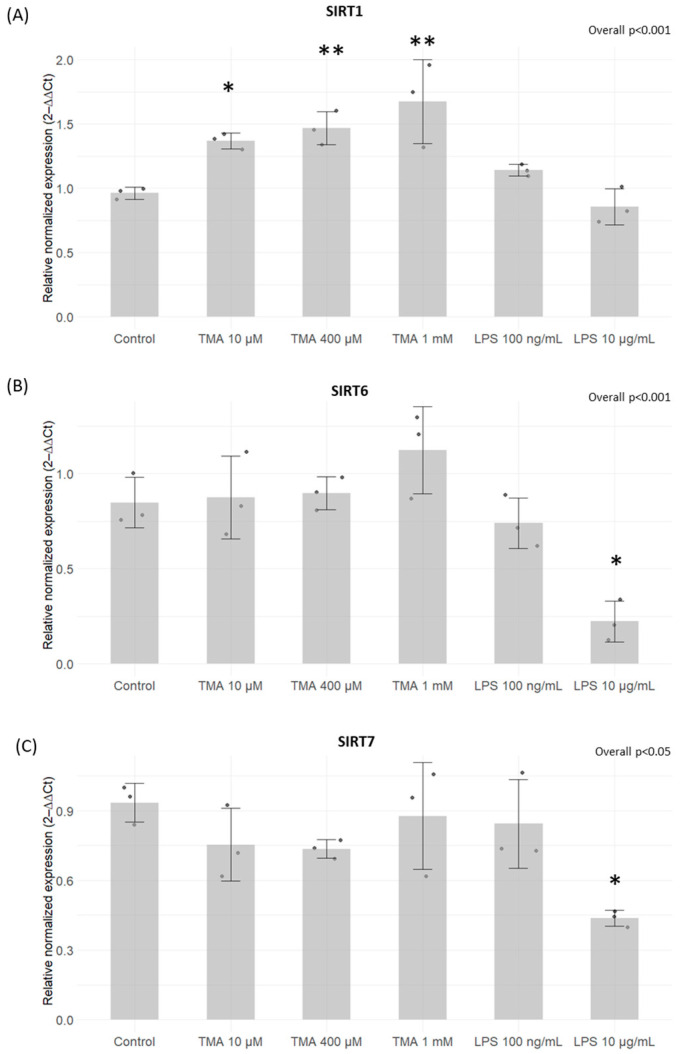
Expression levels of SIRTs. *SIRT1* (**A**), *SIRT6* (**B**), and *SIRT7* (**C**) expression levels on Caco-2 cells after TMA treatments measured with the qPCR. Significantly elevated levels of SIRT1 were measured in Caco-2 cells exposed to TMA at all tested concentrations (**A**). The expression of SIRT6 and SIRT7 was affected only by LPS 10 µg/mL (**B**,**C**). * *p* < 0.05; ** *p* < 0.01 vs. Control.

A significant increase in DNMT1 expression was induced by TMA at 10 µM (*p* < 0.05) and 400 µM (*p* < 0.05) (Figure 3A). Conversely, a statistically significant decrease in DNMT3A expression was observed after exposure for 24 h to TMA at 1 mM (*p* < 0.05) and LPS at 100 ng/mL (*p* < 0.05) (Figure 3B). On the other hand, DNMT3B expression remained unaffected by both TMA and LPS treatments (*p* > 0.05) (Figure 3C). Significantly elevated levels of SIRT1 were measured in Caco-2 cells exposed to TMA at all tested concentrations (10 µM, *p* < 0.05; 400 µM, *p* < 0.01; 1 mM, *p* < 0.01) (Figure 4A). In contrast, the expression of SIRT6 and SIRT7 was affected only by LPS 10 µg/mL (Figure 4B,C).

### 3.4. DNMT and SIRT Enzymatic Activity

**Figure 5:**
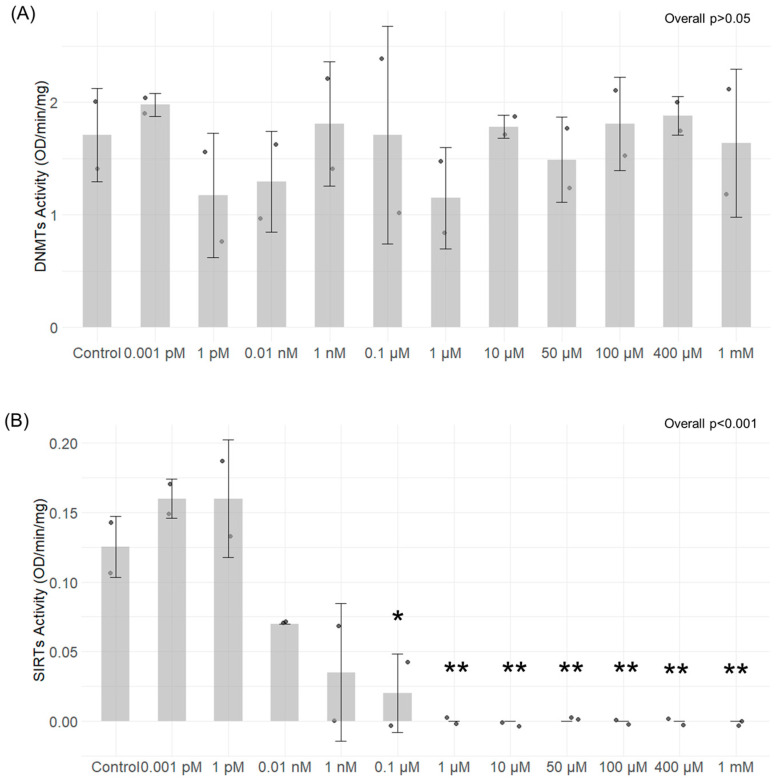
DNMTs and SIRTs enzymatic activity after exposure to different concentrations of TMA. DNMTs activity (**A**) and SIRTs’ activity (**B**) were tested in a cell-free experimental setting after exposure of nuclear extract to different concentrations of TMA using the EpiQuik™ DNA Methyltransferase Activity/Inhibition Assay Kit (EpigenTek, Farmingdale, NY, USA) and the Universal SIRT Activity Assay Kit (Abcam, Waltham, MA, USA), respectively. TMA did not provoke any notable changes in DNMT activity at any of the concentrations tested (**A**). In contrast, the results indicate that TMA can hamper SIRTs’ activity in a concentration-dependent manner; the inhibition of their activity was significant within the concentration range of 0.1 µM to 1 mM (**B**). * *p* < 0.05; ** *p* < 0.01 vs. Control.

The ability of different TMA concentrations to modulate the activity of SIRTs and DNMTs isolated from Caco-2 cells was tested in a cell-free experimental setting. TMA did not elicit any notable changes in DNMT activity at any of the concentrations tested (overall *p* > 0.05) (Figure 5A). Conversely, the results indicate that TMA can hamper SIRTs’ activity in a concentration-dependent manner. The inhibition of their activity was significant within the concentration range of 0.1 µM to 1 mM (overall *p* < 0.001) (Figure 5B).

### 3.5. mtDNA Quantification and Methylation

**Figure 6:**
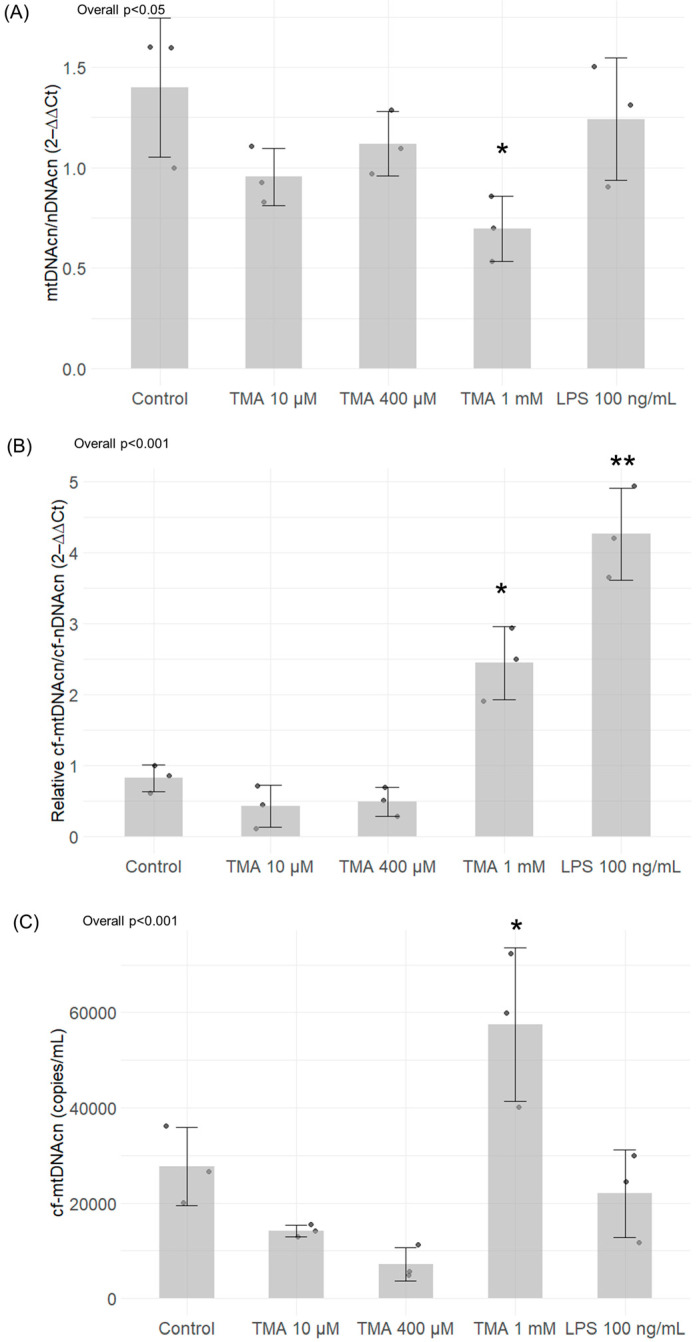
MtDNA quantification in Caco-2 cells exposed to different concentrations of TMA and in the cell culture medium. Intracellular mtDNAcn (**A**) and cf-mtDNA released in the culture medium (**B**,**C**) were assessed by Caco-2 cells after TMA treatments. Panel (**B**) shows values of cf-mtDNAcn normalized for cf-nDNAcn, while panel (**C**) shows the absolute quantification of mtDNAcn/mL of medium. Significant variations in mtDNAcn were observed in the treatment with TMA 1 mM, while neither the lower TMA concentrations nor LPS 100 ng/mL were able to affect intracellular mtDNAcn (**A**). As for the release of mtDNA from the cell following an induction of the inflammatory condition, the relative quantification analysis revealed a significant increase in cf-mtDNA after exposure to 1 mM TMA and LPS 100 ng/mL (**B**). The absolute quantification analysis of cf-mtDNAcn by digital PCR confirmed a significant increase in cf-mtDNA only upon TMA 1 mM treatment (**C**). * *p* < 0.05; ** *p* < 0.01 vs. Control.

The treatment with TMA 1 mM induced a significant decrease in intracellular mtDNAcn (*p* < 0.05) (relative to nDNA copies). Neither the lower TMA concentrations nor LPS 100 ng/mL were able to affect mtDNAcn (Figure 6A). As for the release of mtDNA from the cell following an induction of the inflammatory condition, the relative quantification analysis revealed a significant increase in cf-mtDNA (over cf-nDNAcn) released into the medium by Caco-2 cells after exposure to 1 mM TMA (*p* < 0.05) and LPS 100 ng/mL (*p* < 0.01) (Figure 6B). Of note, the absolute quantification analysis of cell-free mtDNAcn (cf-mtDNAcn) performed by Qiacuity digital PCR confirmed a significant increase in the absolute copies/mL of cf-mtDNA released in the medium only upon TMA 1 mM treatment (*p* < 0.05) (Figure 6C).

The methylation levels were investigated in three areas of mtDNA: the regulatory regions HSP and LSP as well as in the ND6 mitochondrial gene. Methylation levels detected by bisulfite pyrosequencing were very low in all the three areas (around 2–3%). A significant increase in the methylation of the LSP area was measured after TMA 1 mM treatment with respect to controls (*p* < 0.01), with a signal which was almost doubled compared to the control (Supplementary Materials Figure S1A). This increase was driven by the effect of the second CpG (CpG2) analyzed in the LSP area (*p* < 0.001) (Supplementary Materials Figure S1B).

### 3.6. Expression of Mitochondrial Genes

**Figure 7:**
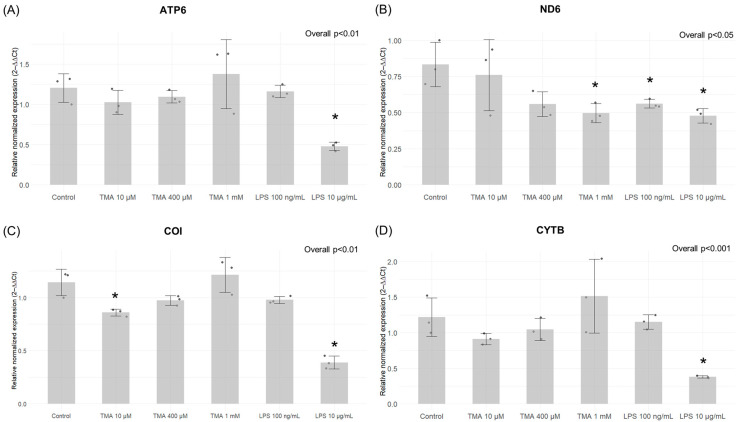
Expression of mitochondrial genes by qPCR. The gene expression of ATP6 (**A**), ND6 (**B**), CO1 (**C**), CYTB (**D**) in the different experimental conditions. The expression levels of ND6 significantly decreased with the highest TMA concentration (TMA 1 mM), as well as both LPS treatments (**B**). Conversely, CO1 showed significant downregulation after treatments with the lowest TMA concentration (TMA 10 µM) and LPS 10 µg/mL (**C**). The expression levels of ATP6 and CYTB were not affected by TMA treatments but only by LPS 10 µg/mL (**A**,**D**). * *p* < 0.05 vs. Control.

The expression levels of ND6 significantly decreased with the highest TMA concentration (TMA 1 mM, *p* < 0.05), as well as both LPS treatments (*p* < 0.05) (Figure 7B). Conversely, CO1 showed significant downregulation after treatments with the lowest TMA concentration (TMA 10 µM, *p* < 0.05) and LPS 10 µg/mL (*p* < 0.05) (Figure 7C). The expression levels of ATP6 and CYTB were not affected by TMA treatments but only by LPS 10 µg/mL (*p* < 0.05 for both genes) (Figure 7A,D).

### 3.7. ATP Quantification

**Figure 8:**
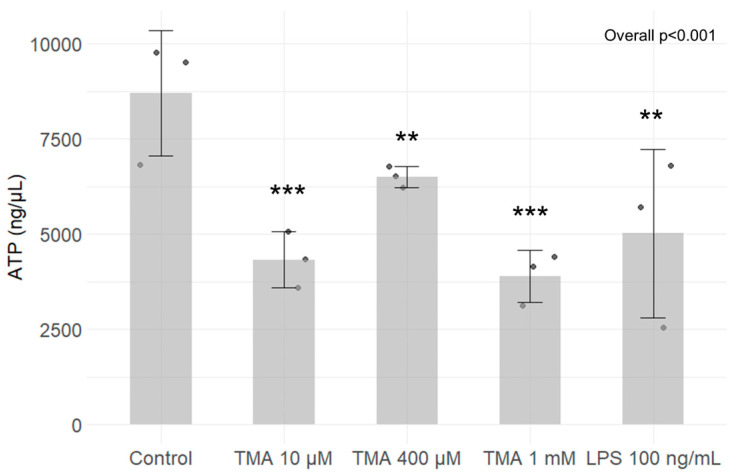
ATP quantification. ATP levels in Caco-2 cells exposed to different TMA concentrations and LPS (as positive control) was measured using the ATP Colorimetric Assay Kit (Sigma-Aldrich, Germany). A decrease in ATP content was observed for all TMA concentrations tested, as well as exposing cells to 100 ng/mL LPS. ** *p* < 0.01; *** *p* < 0.001 vs. Control.

To assess the impact of TMA on intracellular ATP levels, Caco-2 cells were cultured in the presence of different concentrations of TMA (10 µM, 400 µM, and 1 mM). After 24 h, intracellular ATP was quantified on lysed cells. ATP contents is expressed as nmoles ATP per 1 million cells. A decrease in ATP content was observed for all TMA concentrations tested (10 µM TMA, *p* < 0.001; 400 µM TMA, *p* < 0.01 and 1 mM TMA, *p* < 0.001), as well as exposing cells to 100 ng/mL LPS (*p* < 0.01) (Figure 8). As ATP content reflects the metabolic activity of cells, here, we confirm that TMA slows down the cellular metabolism in Caco-2 cells.

### 3.8. Mitochondrial Membrane Potential

The mitochondrial membrane potential (ΔΨm) serves as a crucial gauge not only for mitochondrial activity, indicating ATP production, but also as a marker for overall cellular well-being. In this study, the impact of different doses of TMA on the mitochondrial membrane potential was explored. Following a 24 h exposure to various TMA concentrations, (from 0.01 µM to 1 mM), and to two LPS concentrations (100 ng/mL and 10 µg/mL), no notable changes in the mitochondrial membrane potential were discerned across the tested concentrations (overall *p* > 0.05) (Supplementary Materials Figure S2).

### 3.9. Assessment of the Intestinal Permeability

**Figure 9:**
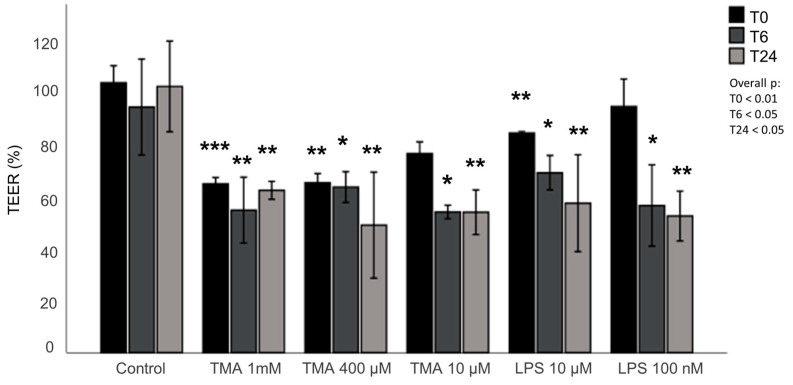
Assessment of the intestinal permeability. The effect of TMA on intestinal barrier was assessed performing TEER measurement in cells cultured in transwells during the treatment with TMA (1 mM, 400 µM, 10 µM) and LPS (10 µg/mL, 100 ng/mL) at time 0, 6 and 24 h post treatment. The values shown in the figure are expressed as a percent of control. * *p* < 0.05; ** *p* < 0.01; *** *p* < 0.001 vs. Control measured at the same time (T0 or T6 or T24, respectively).

The integrity of the intestinal monolayer after 24 h exposure to different concentrations of TMA was investigated using the TEER assay. The epithelium integrity was significantly affected by TMA exposure at time 0, and after 6 h and 24 h, decreasing significantly when compared with the control group at the same time points (Figure 9). Similarly, a significant reduction in the epithelium integrity was measured after exposure to LPS (100 ng/mL, 10 µg/mL), used as positive control for a pro-inflammatory condition (Figure 9). In contrast, no significant differences in permeability were measured between the different treatments with the Lucifer Yellow assay (Supplementary Materials Figure S3). To evaluate the effect of TMA on intestinal permeability, the expression levels of the thigh junctions ZO-1, CLDN1 and OCLN were also measured in Caco-2 cells after the exposure to various concentrations of TMA (10 µM, 400 µM, and 1 mM) and to LPS (100 ng/mL and 10 µg/mL). No significant difference was measured in the expression levels of either ZO-1, CLDN1 or OCLN genes when compared to the control (Supplementary Materials Figure S4).

## 4. Discussion

Numerous studies have proven the existence of a causal link between diet, gut microbiota and human diseases [36]. Addressing the gut microbiota and their metabolites emerges as a promising approach in managing complex multifactorial pathologies, including cardiovascular [37] and metabolic diseases [38,39]. Among diet-derived metabolites suggested as potentially dangerous for the human health, TMAO and TMA have attracted increasing attention [4,40]. As TMAO in the bloodstream is predominantly derived from a nearly complete hepatic oxidation of TMA, most scientific inquiries have focused on TMAO [37,41], with only a limited number of studies exploring the impact of TMA on health. Jaworska et al. indicated that cardiovascular patients exhibit more than double the plasma TMA levels than their healthy counterparts, accompanied by a comparatively smaller disparity in TMAO levels [42]. Altered levels of circulating TMA was also associated to CVD [43]. Despite its systemic role, considering that TMA is produced in the gut, exploring its local impact on the intestinal environment becomes particularly intriguing. The results from our study support the hypothesis that an excess of dietary-derived TMA can affect gut health by modulating intestinal permeability, inflammation and epigenetic homeostasis.

In particular, elevated TMA levels stimulated pro-inflammatory signaling in Caco-2 cells, as evidenced by the heightened expression of pro-inflammatory cytokines IL-6 and IL-1β (Figure 2). The pro-inflammatory effect associated to TMA aligns with prior research [5]. Pro-inflammatory effects were observed at the highest concentration tested (where the increase was higher than in cells exposed to LPS 100 ng/mL) but not at the lowest. This suggests that low and physiological levels of this molecule likely have no adverse effects on the gut, whereas conditions that lead to an excess of TMA may pose a risk due to its pro-inflammatory effects.

Of note, previous findings showed that inflammation has the potential to cause epigenetic alterations [17]. Foran et al. observed that IL-6 increases DNMT1 expression, causing the excessive methylation of tumor suppressor genes [44]. Yang et al. confirmed this observation by showing that IL-6 silences the expression of suppressor of cytokine signaling 3 (SOCS 3) by inducing high expression levels of DNMT1 [45]. Despite the fact that changes in gene expression have been linked to inflammation, the resulting impact on the methylome varies across different contexts, making the overall picture complex. Changes in methylation patterns, as well as disturbances in the function and abundance of SIRTs, have been previously linked to various biological conditions and human disorders, including inflammatory responses [46,47]. In our study, TMA was not able to modulate DNMT activity in cell-free in vitro tests. We found a mild modulation of the expression of genes encoding for DNMTs after their exposure to different TMA concentrations in the cellular model. Despite the fact that our findings suggest the potential ability of TMA to perturb DNMT expression, we could not detect a clear association between inflammation induced by TMA (which was especially observed at a high concentration, i.e., 1 mM) and DNMT expression. In contrast, TMA was able to significantly modulate SIRT activity, with a dose-dependent effect.

Notably, SIRT1 expression was upregulated in Caco-2 cells exposed to the highest dose of TMA (1 mM), suggesting a potential compensatory response to offset the decline in enzymatic activity. Also, the inhibition of SIRT activity might be associated with the TMA’s pro-inflammatory action, given that the suppression of SIRTs is a recognized facet of the acute inflammatory response measured both in laboratory settings and within living organisms [48]. Indeed, NAD^+^-dependent SIRTs play essential, distinct roles in both chronic and acute inflammation, with chronic inflammatory diseases often associated with a deficient “low-sirtuin” state. Maintaining an adaptive phenotype relies on continuous NAD^+^ generation, along with heightened SIRT1 expression and activation [49]. The substantial depletion of NAD^+^ within cells during inflammatory responses [50] could explain the reduction in SIRT activity, as these enzymes depend on intracellular NAD^+^ as a cofactor. Of note, previous evidence showed that TMAO represses SIRT expression [51]. Luo T. et al. [52] documented an upregulation of DNMTs in response to TMAO in a study where mice received TMAO-supplemented water. Additionally, TMAO treatment in RAW264.7 cells was associated with an elevated expression of both DNMT1 and DNMT3B. Altogether, these findings suggest that both TMA and TMAO might affect epigenetic regulations by affecting these pathways. As observed for proinflammatory cytokines, TMA can upregulate the gene expression of DNMT1 (Figure 3) and SIRT1 (Figure 4) more than what is observed with LPS. This suggests that alterations of these parameters are driven by TMA specifically, rather than by inflammation per se. However, while we can speculate that TMA may directly affect Sirtuin homeostasis, as indicated by its ability to suppress Sirtuins’ activity (Figure 5), we cannot draw the same conclusion for DNMT1. Further studies are needed to determine whether TMA directly impacts DNMT1 expression independently of inflammation, especially considering the central role of this enzyme in maintaining overall DNA methylation patterns within the cell. Considering the widely recognized involvement of mitochondria in inflammation, we tested if intestinal cells exposed to different TMA concentrations undergo changes in mitochondrial functions. The capacity of TMAO to alter the mitochondrial energy metabolism has been previously demonstrated in cardiac tissues [53]. Previous evidence also showed that administration of TMAO into WT mice lead to a reduction in ATP levels in cardiac tissue throughout the suppression of mitochondrial complex IV activity [54]. However, little is known about the action of TMA on the subunits of the respiratory chain. In our study, TMA induced a decrease in intracellular ATP concentration. This evidence is in line with the results revealed by Jalandra et al. [5]. Despite the effect of TMA on ATP levels, no significant alteration of the mitochondrial membrane potential after TMA treatments was measured. Nevertheless, the gene expression of CO1 and ND6, two components of the electron transport chain, is downregulated by TMA. CO1 and ND6 have a crucial role in the process of energy homeostasis and the decrease in their expression caused by TMA treatment corroborates the hypothesis of a direct TMA-induced mitochondrial dynamics damage. The mechanism of regulation of the mitochondrial gene expression has not been totally elucidated yet: whether it is regulated by the methylation on the mtDNA is still a matter of debate. In our experimental settings, we measured by bisulfite pyrosequencing low levels of mtDNA methylation in the light strand promoter region (LPS), which doubled in cells exposed to the highest dose of TMA (1 mM). Alterations of DNA methylation in this area, in association with a decreased expression of the ND6 gene, suggest a potential correlation between these phenomena (in particular, considering that ND6 is the only gene under the control of LSP promoter). Of note, previous evidence demonstrated that higher ND6 methylation levels were associated with lower ND6 expression in a liver sample of patients with Metabolic Steatohepatitis (MeSH) and in leukocytes of diabetic patients [10,55,56]. Although intriguing, further studies are necessary to confirm this preliminary evidence and clarify the role of mtDNA methylation in this context.

Remarkably, we found that TMA has a significant impact on mtDNAcn, both in the intracellular and extracellular context. In our study, Caco-2 cells exposed to TMA 1 mM showed lower levels of intracellular mtDNAcn over nDNA copies. The mtDNAcn has been considered a biomarker of mitochondrial health: a high mtDNAcn level has been hypothesized to reflect a proper mitochondrial DNA translation leading to mitochondrial genome stability, while a decrease in mtDNAcn has been associated with the downregulation of mitochondrial transcription and a decline in OXPHOS proteins levels [7]. This evidence corroborates the hypothesis of a detrimental effect of TMA on mitochondrial functions. The potential alteration of mtDNA functions is reflected also in the increased cf-mtDNA measured in the medium of cells exposed to 1 mM TMA. Cf-mtDNA has been suggested to be released by cells throughout cellular death/breakdown processes or throughout an active regulated process probably linked to inflammation [57]. Indeed, cf-mtDNA has been found increased in plasma of inflammatory pathologies patients, even though the mechanistic explanation for this association has not been elucidated yet [29,58,59]. In vitro studies showed that the treatment of cells with purified mtDNA and proinflammatory compounds trigger pro-inflammatory responses [57]. In particular, the co-stimulation of monocytes with purified cf-mtDNA and LPS potentiated the pro-inflammatory action of LPS [57,60]. Furthermore, the combination of mtDNA and N-Formylmethionyl-leucyl-phenylalanine (fMLS) in polymorphonuclear leukocytes induced IL-8 more than fMLF alone [57,61]. Thus, we can speculate that the released cf-mtDNA might foster the local pro-inflammatory action of TMA in the gut. Of note, while both TMA 1 mM and LPS were associated to increased mtDNAcn/nDNAcn ratio, the absolute quantity of cf-mtDNA released into the medium measured by digital PCR was higher in cells exposed to TMA compared to LPS. This finding suggests that TMA has a specific effect on mitochondrial dynamics, which aligns with our additional results.

Our findings also suggest that exposure to TMA can damage the intestinal epithelium integrity, potentially promoting a leaky gut, which is often associated with a pro-inflammatory condition. Although preliminary, this result supports further studies investigating how factors that increase gut TMA levels (including dietary precursors and microbiome composition) affect intestinal barrier functions and possibly contribute to leaky gut.

This study has several limitations. Firstly, we were able to perform the experiment only in vitro and in a singular cell line. Further investigations aimed at confirming these preliminary findings in other cell lines and in vivo are necessary before addressing the TMA of potentially harmful effects and driving therapeutic or dietetic intervention. Also, despite the fact that Caco-2 cells are a widely used to generate a surrogate of intestinal epithelium and testing diet-derived molecules, we have to keep in mind that they are a tumoral cell line, with potential responses that might not be replicated in more physiological conditions. Similar studies on different cellular models (non-transformed cells or organoids) that might be able to simulate better physiological condition of healthy humans are warranted. Also, given the potential effect of TMA on mitochondrial homeostasis, further investigations showing the impact of TMA on mitochondrial morphology and functionality (e.g., respiration measured with Seahorse Analyzer or Oroboros O2k) would add valuable insights to this complex picture. Finally, further studies are needed to assess the physiological levels of TMA in the gut to determine the concentrations to which the gut can be exposed in real-life contexts. This is crucial due to the significant variability in TMA levels, which are directly influenced by diet and microbiome composition.

Despite these limitations, our study provides preliminary evidence that TMA excess in the gut might not only endorse an increase in circulating TMAO levels but also promote in situ inflammation, disrupting both epigenetic and mitochondrial homeostasis. Despite the fact that alterations induced by TMA in the conditions we tested did not induce a complete disruption of cellular functions, a prolonged activation of the pro-inflammatory state induced by TMA may lead to increased intestinal permeability and compromised mitochondrial functions. Further studies corroborating these preliminary findings in different experimental context could prompt a reassessment of dietary recommendations concerning TMA precursors and microbiome modulation strategies designed to reduce TMA accumulation and its potential adverse effects.

## Data Availability

The authors confirm that the data supporting the findings of this study are available within this article and its Supplementary Materials.

## Supplementary Materials

The following supporting information can be downloaded at: https://www.mdpi.com/article/10.3390/antiox13091061/s1. Figure S1: Evaluation of mtDNA methylation by bisulfite pyrosequencing; Figure S2: Evaluation of mitochondrial membrane potential; Figure S3: Assessment of the intestinal permeability; Figure S4: Expression levels of tight junctions; Table S1: Primer sequences used for gene expression analysis.

## Abbreviations

AP: apical; ATP6: ATP synthase membrane 6; B2M: Beta 2 microglobulin; BL: basal; BSA: bovine serum albumin; Caco-2 cells: colon adenocarcinoma cells; Cf-DNA: cell-free DNA; Cf-mtDNA: cell-free mitochondrial DNA; Cf-mtDNAcn: cell-free mitochondrial DNA copy number; Cf-nDNA: cell-free nuclear DNA; CO1: cytochrome c oxidase 1; CVDs: cardiovascular diseases; CYTB: cytochrome B; D-loop: displacement loop; DMEM: Dulbecco’s modified Eagle’s medium; DNMT1: DNA methyltransferase 1; DNMT3A: DNA methyltransferase 3A; DNMT3B: DNA methyltransferase 3B; DNMTs: DNA methyltransferases; FBS: fetal bovine serum; fMLS: N-Formylmethionyl-leucyl-phenylalanine; FMOs: hepatic flavin monooxygenases; HSP: promoter of the heavy strand; IL-1β: Interleukin 1β; IL-6: Interleukin 6; LPS: lipopolysaccharide; LSP: promoter of the light strand; LY: Lucifer Yellow; MeSH: Metabolic Steatohepatitis; mtDNA: mitochondrial DNA; mtDNAcn: mitochondrial DNA copy number; MTT: 3-(4,5-Di-2-yl)-2,5-ditetrazolium bromide; ND6: NADH dehydrogenase 6; NEAAs: non-essential amino acids; NUMTs: nuclear insertions of mitochondrial origin; PBS: Phosphate-Buffered Saline; qPCR: quantitative real-time polymerase chain reaction; SIRT1: Sirtuin 1; SIRT6: Sirtuin 6; SIRT7: Sirtuin 7; SIRTs: Sirtuins; SOCS 3: cytokine signaling 3; TEER: transepithelial electrical resistance; TMA: trimethylamine; TMAO: Trimethylamine-N-Oxide; ΔΨm: mitochondrial membrane potential.
