# Supplementary material for: Gut Microbiota-Derived Trimethylamine Promotes Inflammation with a Potential Impact on Epigenetic and Mitochondrial Homeostasis in Caco-2 Cells
Source: Antioxidants (Basel). 2024 Aug 30;13(9):1061. doi: 10.3390/antiox13091061 (PMC11428692; doi:10.3390/antiox13091061)

Supplementary materials

Supplementary Table S1. Primer sequences used for gene expression analysis.

	Fw (5'-3')	Rv (5'-3')	RefSeq accession number
SIRT1	ACGCTGGAACAGGTTGCGGG	AGCGGTTCATCAGCTGGGCAC	NM_001314049.2
SIRT6	AGTTCGACACCACCTTTGAG	CGTACTGCGTCTTACACTTG	XM_054321190.1
SIRT7	CGTCCGGAACGCCAAATAC	GACGCTGCCGTGCTGATT	XM_054316405.1
IL-6	TGCAATAACCACCCCTGACC	GTGCCCATGCTACATTTGCC	XM_054358146.1
IL-1β	AGATGATAAGCCCACTCTACAG	ACATTCAGCACAGGACTCTC	XM_047444175.1
ND6	CAAACAATGTTCAACCAGTAACCACTAC	ATATACTACAGCGATGGCTATTGAGGA	NM_005006
CYTB	ATCACTCGAGACGTAAATTATGGCT	TGAACTAGGTCTGTCCCAATGTATG	NM_001031702
CO1	GACGTAGACACACGAGCATATTTCA	AGGACATAGTGGAAGTGAGCTACAAC	NM_001114.4
ATP6	TAGCCATACACAACACTAAAGGACGA	GGGCATTTTTAATCTTAGAGCGAAA	NM_001686.4
ZONULIN	TTCACGCAGTTACGAGCAAG	TTGGTGTTTGAAGGCAGAGC	XM_054378717.1
OCCLUDIN	GGGCATTGCTCATCCTGAAG	GCCTGTAAGGAGGTGGACTT	XM_054351382.1
CLAUDIN	TGGTCAGGCTCTCTTCACTG	TTGGATAGGGCCTTGGTGTT	NM_021101.5
DNMT1	Biorad PrimePCR™ SYBR® Green Assay: DNMT1, Human Unique Assay ID:qHsaCED0044343		NM_001130549.2
DNMT3A	Biorad PrimePCR™ SYBR® Green Assay: DNMT3a, Human Unique Assay ID: qMmuCED0037493		NM_006892.5
DNMT3B	Biorad PrimePCR™ SYBR® Green Assay: DNMT3b, Human Unique Assay ID: qHsaCED0042577		NM_006892.4

Supplementary Figure S1. Evaluation of mtDNA methylation by bisulfite pyrosequencing. Percentage of average methylation of the LSP area (A) and focus on the second CpG (CpG2) of the LSP area (B) for the different treatments. A significant increase in the methylation of the LSP D-Loop area has been measured after TMA 1mM treatment (A); this increase was driven by the effect of the CpG2 analysed in the LSP D-Loop area (B). **p <0.01; ***p <0.001***p <0.001 vs Naif.

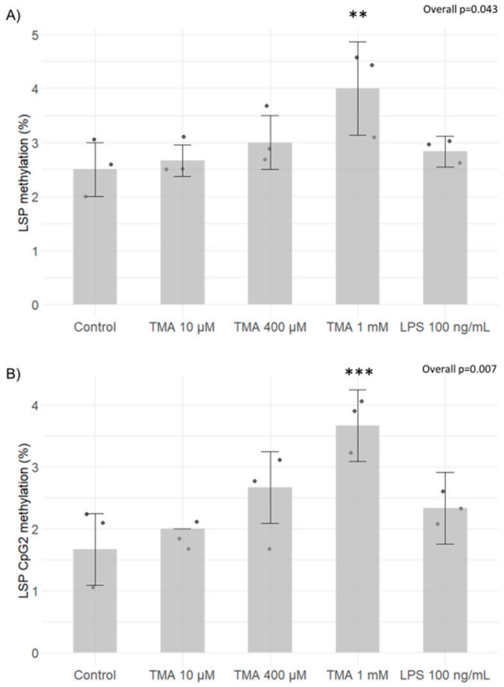

Supplementary Figure S2. Evaluation of Mitochondrial membrane potential. Mitochondrial membrane potential (ΔΨm) in Caco-2 cells after 24h TMA treatments. The analysis was conducted in technical duplicates.

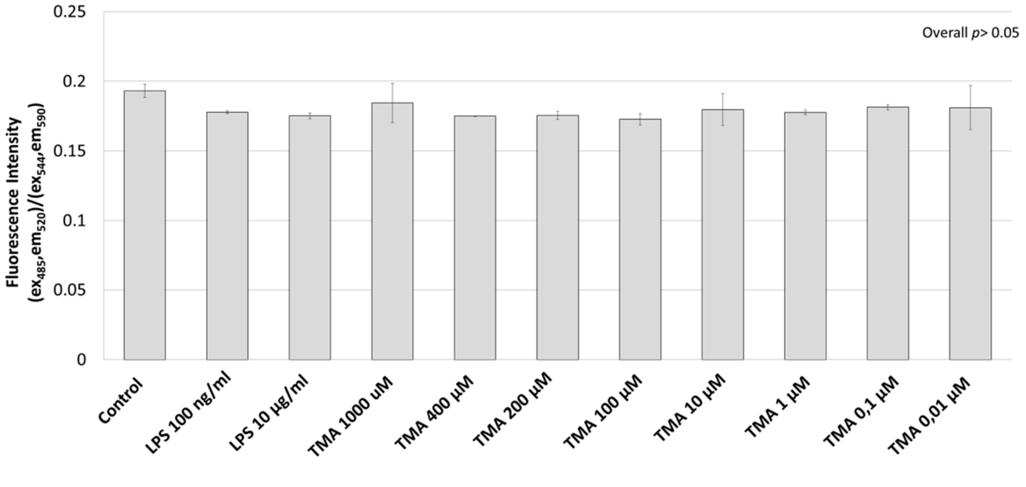

Supplementary Figure S3. Assessment of the intestinal permeability. Intestinal permeability of Caco-2 cells epithelium model measured by the Lucifer Yellow (LY) assay. The plot shows the permeability of the epithelium at time 0, 30, 60 and 120 minutes after the addition of the LY on the AP compartment. The analysis was conducted in duplicates.

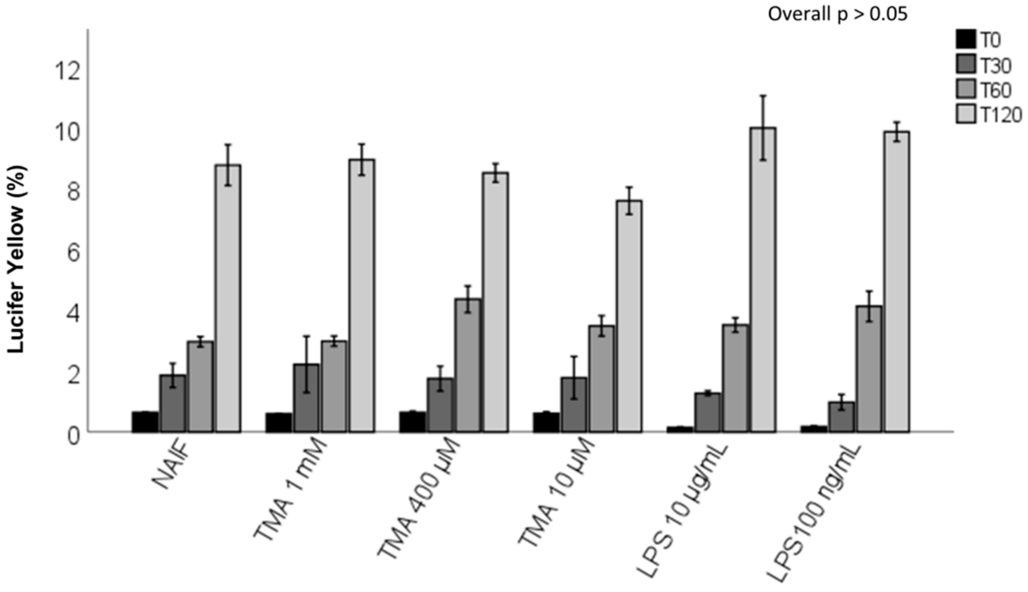

Supplementary Materials Figure S4. Expression levels of tight junctions. Expression levels of ZO-1 (A), OCLN (B) and CLDN1 (C) in Caco-2 cells treated with TMA (10µM, 400µM, and 1mM), and LPS (100 ng/ml and 10 µg/ml) was measured by qPCR. No significant difference was observed in the expression levels of either ZO-1, OCLN or CLDN1 genes when compared to the control.

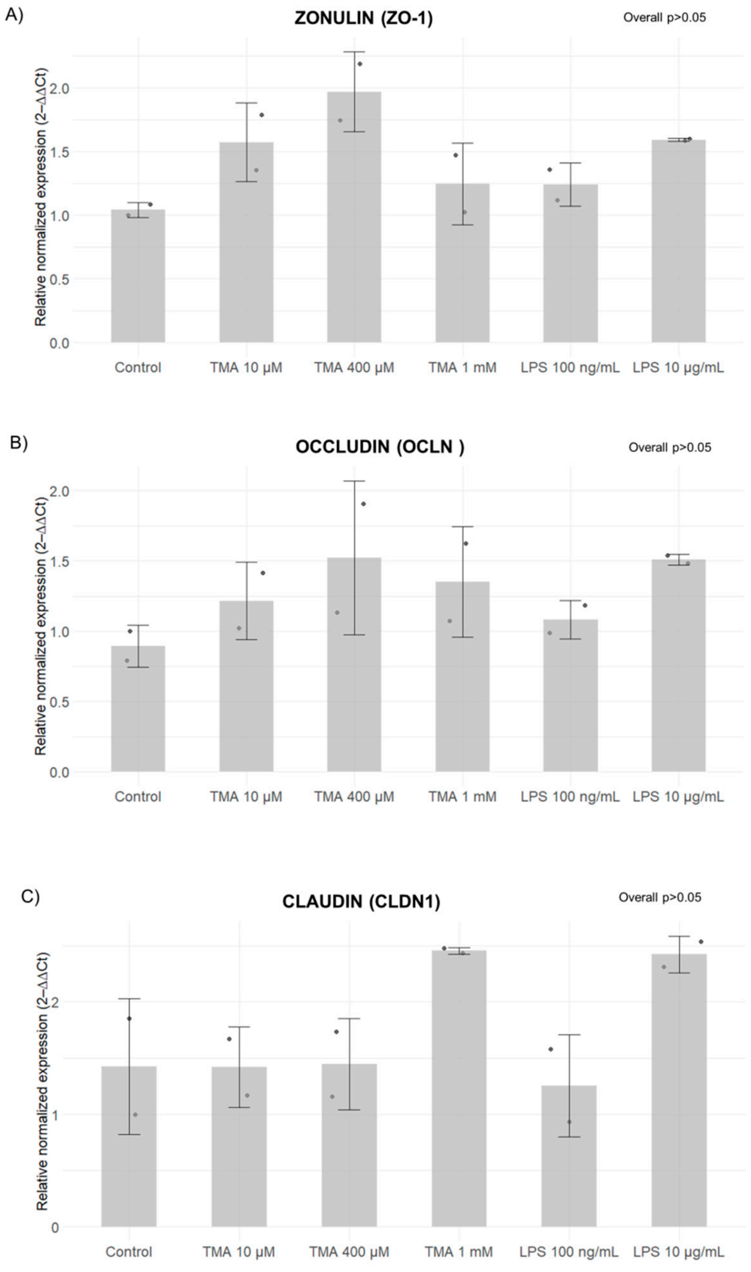

Supplement: Supplementary file 1 [file antioxidants-13-01061-s001.zip › antioxidants-3165011-supplementary.pdf]
